# CoQ10-Supported HIIT Modulates Skeletal Muscle and Hippocampal Biomarkers in Rats: A Randomized, Repeated-Measures, Post-Test Controlled Design

**DOI:** 10.3390/antiox14111360

**Published:** 2025-11-14

**Authors:** Büşra Yılmaz, Ömer Şenel, Ayşen Çalıkuşu, Elif Gülçiçek Abbasoğlu, Yavuz Yasul, Elvan Anadol, Fatih Sarısoy, Kerem Atalar, Meltem Bahçelioğlu, Canan Yılmaz

**Affiliations:** 1 Faculty of Sport Sciences, Gazi University, Teaching Staff and Classroom Building (BLOCK D) Emniyet Neighborhood, Abant-1 Street, No: 10/1D, Yenimahalle, 06560 Ankara, Türkiye; busra.yuce.87@gmail.com (B.Y.); fatihsarisoy08@gmail.com (F.S.); 2 Faculty of Medicine, Department of Anatomy, Ege University, 35100 Izmir, Türkiye; aysencalikusu01@gmail.com; 3 Neuroscience and Neurotechnology Center of Excellence (NÖROM), Gazi University, 06560 Ankara, Türkiye; eliftopa@gazi.edu.tr (E.G.A.); kerematalar@gazi.edu.tr (K.A.); meltemb@gazi.edu.tr (M.B.); 4 Bafra Vocational School, Ondokuz Mayıs University, 55270 Samsun, Türkiye; yavuz.yasul@omu.edu.tr; 5 Laboratory Animals Breeding and Experimental Research Center (GUDAM), Gazi University, 06560 Ankara, Türkiye; elvanadol@gazi.edu.tr; 6 Faculty of Medicine Basic Medical Sciences, Medical Biochemistry, Gazi University, 06560 Ankara, Türkiye; cananyilmaz@gazi.edu.tr

**Keywords:** BDNF, coenzyme Q10, citrate synthase, GFAP, lactate threshold, HIIT, irisin

## Abstract

This study examined how coenzyme Q10-supported high-intensity interval training (HIIT) influences plasma lactate threshold, skeletal muscle oxidative capacity, circulating irisin and corticosterone, and hippocampal brain-derived neurotrophic factor (BDNF) and glial fibrillary acidic protein (GFAP) levels in rats. Forty-eight male Sprague Dawley rats (8 weeks old; 250.4 ± 11.2 g) were randomized into four groups: control (C), coenzyme Q10 (Supp), HIIT, and HIIT with coenzyme Q10 (HIITsupp). HIIT was performed five days per week on a treadmill following a four-stage familiarization. Coenzyme Q10 (5 mg/kg/day) was given by gavage 30 min before HIIT during weeks II–IV. Plasma lactate threshold, corticosterone, irisin, and citrate synthase (CS) activity were measured by ELISA, while hippocampal BDNF and GFAP were analyzed by both ELISA and immunohistochemistry. The HIITsupp group showed greater muscle mass, CS activity, plasma irisin, and hippocampal BDNF, along with lower GFAP and lactate threshold than the C, Supp, and HIIT groups. The Supp group had the lowest corticosterone, while the HIIT group maintained the highest lactate threshold before supplementation. Principal Component Analysis (PCA) indicated distinct clustering, with the C group closely associated with GFAP and corticosterone, whereas the HIITsupp group aligned with oxidative and neurotrophic markers. Coenzyme Q10-supported HIIT improved muscle oxidative capacity, lowered lactate, and modulated corticosterone, GFAP, and hippocampal BDNF, indicating integrated metabolic and neurobiological adaptations.

## 1. Introduction

Exercise elicits comprehensive adaptive responses in human health by modulating multiple biological processes such as energy metabolism, mitochondrial function, and neuroplasticity. High-intensity interval training (HIIT) stands out as one of the most potent stimuli for these adaptations, generating more marked cellular and molecular changes than conventional endurance exercise, due to its brief duration combined with maximal exertion [1]. The increased energy demand during HIIT activates glycolytic metabolism in muscle cells, leading to elevated lactate production [2]. Circulating lactate is transported to brain tissue via monocarboxylate transporters (MCTs), where it modulates energy metabolism through the GPR81 receptor. Additionally, it activates the AMPK and SIRT1 signaling pathways, thereby enhancing the expression of PGC-1α and FNDC5, playing pivotal roles in mitochondrial biogenesis and metabolic adaptation [3]. The proteolytic cleavage of FNDC5 releases irisin, a myokine capable of crossing the blood–brain barrier and promoting the synthesis of brain-derived neurotrophic factor (BDNF). Irisin-induced upregulation of hippocampal BDNF plays a critical role in synaptic remodeling, neuronal resilience, and the enhancement of cognitive functions [4]. Elevated lactate levels during HIIT have been demonstrated to stimulate irisin release, thereby activating the FNDC5/BDNF axis, which is considered one of the key mechanisms underlying the neurotrophic effects of HIIT [5]. Increased BDNF levels enhance synaptic plasticity, reshape hippocampal neural networks, and support cognitive performance [6,7]. The neurotrophic response to HIIT may vary according to protocol-specific parameters, including duration, intensity, recovery intervals, and individual metabolic variability. However, the optimal HIIT configuration for reliably inducing neuroplasticity has yet to be clearly established [8].

Glial cells act as key modulators of exercise-induced effects on the central nervous system, with astrocytes and microglia playing essential roles in the regulation of synaptic signaling and neuroinflammatory processes [9]. The expression of glial fibrillary acidic protein (GFAP), predominantly in astrocytes, serves as a well-established biomarker reflecting changes in glial morphology, inflammatory activity, and gliotransmitter release. Nevertheless, the influence of nutraceutical strategies combined with HIIT on glial dynamics and GFAP expression remains poorly characterized [10,11].

Coenzyme Q10 (CoQ10), a fundamental cofactor in mitochondrial energy metabolism, is increasingly recognized as a pivotal modulator of mitochondrial function. By facilitating electron transfer between complexes I and III within the electron transport chain, CoQ10 supports ATP synthesis while concurrently mitigating the generation of reactive oxygen species (ROS) [12]. In addition, it activates the transcription factor Nrf2, thereby upregulating the expression of key antioxidant genes such as HO-1 and NQO1, ultimately attenuating oxidative stress–induced neuroinflammation [13]. Evidence from an animal study indicates that CoQ10 enhances hippocampal BDNF expression, preserves synaptic integrity, and reduces neuronal loss. When administered in conjunction with HIIT, CoQ10 has been reported to potentiate mitochondrial function, diminish oxidative stress, and consequently elevate circulating levels of irisin and BDNF [5]. Although previous studies have independently assessed parameters such as citrate synthase activity, irisin, corticosterone, BDNF, and GFAP, the present work offers an integrative approach by concurrently evaluating these metabolic and neurotrophic markers [14,15,16]. This design allows a comprehensive assessment of the coordinated effects of CoQ10-supported HIIT on mitochondrial function, metabolic efficiency, and hippocampal plasticity.

Peripheral adaptations in skeletal muscle are closely interconnected with central nervous system responses. HIIT has been shown to increase citrate synthase (CS) activity in muscle tissue [17]. As the rate-limiting enzyme of the tricarboxylic acid (TCA) cycle, CS catalyzes the condensation of acetyl-CoA and oxaloacetate to initiate mitochondrial energy production. CS activity is widely regarded as a biochemical marker of mitochondrial density, oxidative capacity, and endurance performance [18]. Enhanced CS activity facilitates the oxidative metabolism of pyruvate, thereby limiting lactate accumulation. This elevation in lactate threshold not only improves muscular efficiency but also contributes to more effective activation of the FNDC5/irisin–BDNF axis. Under such high metabolic demand, CoQ10 supplementation supports more efficient energy metabolism by reducing electron leakage and ROS generation within the mitochondria [19,20].

Elevated corticosterone levels during high-intensity exercise may suppress hippocampal BDNF expression and alter glial cell responses [21]. However, when exercise protocols are appropriately structured, these inhibitory effects can be counterbalanced [22]. Previous studies have reported that increases in corticosterone may reduce glial activity and subsequently influence the expression of GFAP [23,24].

This study hypothesizes that HIIT will enhance mitochondrial oxidative capacity in skeletal muscle by increasing citrate synthase activity, which in turn will elevate plasma lactate and irisin levels, thereby augmenting hippocampal BDNF expression. It is further hypothesized that the metabolic stress and corticosterone response elicited by HIIT can be attenuated through CoQ10, which may accelerate lactate clearance, suppress neuroinflammation, and support muscle oxidative capacity.

## 2. Materials and Methods

### 2.1. Experimental Design

This study employed a randomized, repeated-measures, post-test controlled experimental design. Forty-eight male Sprague Dawley rats (8 weeks old; mean body mass 250.4 ± 11.2 g) were used. The experimental procedures were conducted at the Laboratory Animal Breeding and Experimental Research Center of Gazi University, in accordance with the NIH Guide for the Care and Use of Laboratory Animals. Ethical approval was obtained from the Gazi University Animal Experiments Ethics Committee (Approval No: G.Ü. ET: E-6633047-604.01.02-552613). Sample size was determined using G-Power software 3.1, with 5% type I error rate (α = 0.05), 80% power (1 – β = 0.80), an effect size of 2.18, and a two-tailed alternative hypothesis (H1), indicating a minimum requirement of 12 rats per group. Accordingly, the animals were randomly assigned to four groups: control (C), CoQ10 (Supp), high-intensity interval training (HIIT), and HIIT combined with CoQ10 (HIITSupp). Rats were housed under standard conditions (22–25 °C, 12 h light/12 h dark cycle, 55% ± 10% humidity) with three animals per cage. The C group received no intervention. The HIIT protocol was conducted on a flat motorized treadmill (Columbus Instruments Exer-6M Treadmill, USA) five days per week following a four-stage familiarization period.

**Figure 1:**
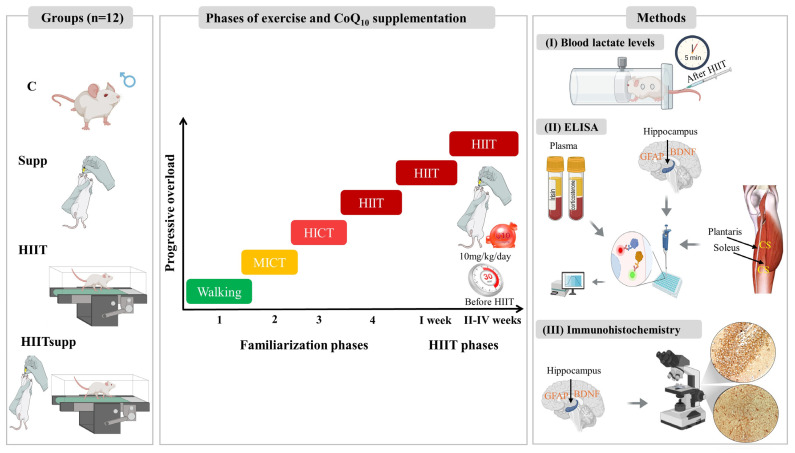
Schematic representation of the experimental design, including familiarization and HIIT phases, CoQ10 supplementation (timing and dosage), and outcome assessments. Blood lactate levels were measured 5 min post-HIIT (I). ELISA analyses were performed to plasma irisin and corticosterone, as well as GFAP, BDNF in hippocampal homogenates, and citrate synthase (CS) activity in skeletal muscle (plantaris and soleus) homogenates (II). Immunohistochemistry was used to detect GFAP and BDNF expression in hippocampal sections (III). The experimental groups were as follows: C: control group; Supp: CoQ10 group; HIIT: high-intensity interval training; HIITsupp: high-intensity interval training combined with CoQ10 group. MICT: moderate-intensity continuous training; HICT: high-intensity continuous training; BDNF: brain-derived neurotrophic factor; GFAP: glial fibrillary acidic protein.

Following completion of the experimental period, the rats were euthanized under ketamine (45 mg/kg, i.m.) and xylazine (5 mg/kg, i.m.) anaesthesia. Blood samples were drawn intracardially into EDTA tubes containing aprotinin and centrifuged at 4.000 rpm for 5 min at 4 °C to obtain serum. Serum corticosterone and irisin concentrations were quantified using commercial kits (SunRedBio, Shanghai, China) with a sandwich ELISA method. Hippocampal tissues from six randomly selected rats in each experimental group were analyzed by ELISA to determine BDNF and GFAP levels. Hippocampal samples from the remaining six rats were used for immunohistochemical examinations. Soleus and plantaris muscles were subjected to ELISA-based assays to assess CS activity and other biochemical parameters. Lactate levels were measured from blood samples obtained 5 min post-exercise (HIIT phase) from six randomly selected animals in each group, which were transferred into capillary tubes (Safeclinitubes; 942-896-D957P-70-100) and analyzed using a Radiometer 900 automated analyzer (Figure 1).

### 2.2. Feeding and Supplement Protocol

Experimental groups were fed a standard rat diet ad libitum. The diet contained 21% protein, 5% fat, and 55% carbohydrate, balanced with essential vitamins and minerals. CoQ10 (Biosynth, CAS No: 303-98-0, FC20535, Berkshire, UK) was administered to the supplemented groups (Supp and HIITsupp) via oral gavage at a daily dose of 5 mg/kg [25]. The Supp group received supplementation alone, whereas the HIITsupp group received the same dose of CoQ10 combined with the high-intensity interval training protocol. To ensure optimal bioavailability, supplementation was administered 30 min prior to each exercise session [26].

### 2.3. Treadmill-Based Exercise Protocol

Exercise groups underwent a structured four-stage familiarization period based on the principles of progressive loading, consisting of treadmill familiarization (walking), moderate-intensity continuous training (MICT), high-intensity continuous training (HICT), and transition to HIIT (Figure 1). The walking, MICT, and HICT stages each lasted 20 min, with speeds set at 0.3 km/h, 0.9 km/h, and 1.5 km/h, respectively [27,28]. The fourth stage involved HIIT-specific running sessions incorporating speed progression and defined running-to-rest ratios. In this stage, rats performed two repetitions of 20-s high-intensity runs at speeds ranging from 30 to 40 m/min, arranged in two sets. Rest intervals between repetitions were 120–150 s (1:4–1:5 running-to-rest ratio), and between sets were 3–4 min (1:9–1:12 running-to-rest ratio).

Following the fourth stage, a HIIT protocol was implemented. During the first week, rats performed three sets of two repetitions, with each repetition consisting of a 20-s run at 30–40 m/min. Rest intervals were 120–150 s between repetitions and 3–4 min between sets. CoQ10 supplementation was not provided during this week. From the second week onward, CoQ10 was administered to the HIITsupp and Supp groups via oral gavage 30 min before exercise. Training load was progressively increased by raising the number of repetitions and sets: three in week 2, four in week 3, and five repetitions in week 4. The number of sets was kept constant at four. Throughout the training period, running speed (30–40 m/min), interval duration (30 s), and rest intervals (120–150 s between repetitions; 3–4 min between sets) remained constant [29]. The sessions were performed under standard environmental conditions at 10:30 a.m. on weekdays.

### 2.4. ELISA

*CS activity* was measured in soleus and plantaris muscle tissues isolated following sacrifice. Samples were stored at −80 °C and thawed on the day of analysis. CS activity was quantified using the Elabscience Citrate Synthase Activity Assay Kit (Cat. No: E-BC-K178-M, Elabscience, Wuhan, China) in accordance with the manufacturer’s instructions. Approximately 20 mg of tissue was homogenized in 180 μL of extraction buffer (1:10, *w*/*v*) at 4 °C, followed by centrifugation at 10.000× *g* for 15 min. Both CS activity and total protein concentration were determined from the supernatant, with enzyme activity expressed as U/g protein and interpreted as a biochemical marker of mitochondrial aerobic capacity in skeletal muscle [30,31]. *BDNF* and *GFAP* levels were determined from hippocampal tissues of six randomly selected rats per group. Samples were homogenized in 14% KCl buffer and centrifuged at 7.000 rpm for 5 min at 4 °C, and the resulting supernatants were analyzed using BT-Lab ELISA kits (Cat. No: E0476Ra, Shanghai, China) in accordance with the manufacturer’s protocol. Streptavidin–HRP secondary antibodies and washing steps were performed using a BIO-TEK ELX50 system, and absorbance was read at 450 nm with a BIO-TEK ELX800-Aotu microplate reader (Winooski, VT, USA). Concentrations were calculated from standard curves and expressed as pg/mg protein. *Corticosterone and irisin* measurements were performed using intracardiac blood samples, transferred to gel-containing yellow-cap tubes, and centrifuged at 3.500 rpm for 10 min to obtain serum. Corticosterone (Cat. No: 201-11-0497) and irisin (Cat. No: 201-11-5196) concentrations were quantified using sandwich ELISA kits from SunRedBio (Shanghai, China). Washing procedures were performed with a BIO-TEK ELX50 system, and absorbance was measured at 450 nm using a BIO-TEK ELX800-Aotu microplate reader. Results were expressed in ng/mL according to the manufacturer’s instructions. Finally, *total protein concentration* was quantified for subsequent normalization of all measured values from muscle and hippocampal samples using the BCA Protein Assay Kit (Cat. No: SK3021, Bio Basic, Markham, ON, Canada). This colorimetric assay is based on the reduction of Cu^2+^ to Cu^+^ by peptide bonds in proteins under alkaline conditions, followed by the formation of a purple complex with bicinchoninic acid (BCA). Absorbance was measured at 562 nm, and concentrations were calculated from a standard curve and expressed in mg/mL.

### 2.5. Immunohistochemistry

Animals were weighed for accurate anesthetic dosing, then deeply anesthetized with ketamine (60 mg/kg, Pfizer) and xylazine (8 mg/kg, Bayer) before humane euthanasia. Following transcardial perfusion with heparinized saline and 4% paraformaldehyde (PFA), brains were post-fixed for 48 h, dehydrated, paraffin-embedded, and sectioned (4 μm) onto charged glass slides. Sections were deparaffinized, rehydrated, and subjected to antigen retrieval (microwave, citrate buffer, pH 6.0, 10 min). Endogenous peroxidase was blocked with 0.3% H_2_O_2_, followed by universal blocking (Thermo Scientific, Waltham, MA, USA). Primary antibodies, anti-BDNF (E-AB-18244, Elabscience, Houston, TX, USA, 1:1000) and anti-GFAP (E-AB-70040, Elabscience, Houston, TX, USA, 1:1000), were incubated overnight (4 °C). After washing, sections were treated with biotinylated secondary antibody, streptavidin–peroxidase, and developed with DAB (0.5 mg/mL). Tissues were dehydrated and coverslipped and imaged at 20×/40× magnifications (Zeiss Axio Scope 5). Histological analyses were performed using a Leica DM 4000B microscope with LAS v4.12 software. Hippocampal subregions (CA1, CA3, DG) were identified using the Rat Brain Atlas [32]. Images were analyzed in ImageJ (v1.54d) with hematoxylin and DAB channels separated digitally. For BDNF, “H-scores = (3 × strong) + (2 × moderate) + (1 × weak) + (0 × negative)” quantified immunoreactivity. For GFAP, DAB-positive cells were counted and expressed as a percentage of total cells per unit area.

### 2.6. Statistical Analyses

Statistical analyses were conducted using IBM SPSS 21.0 Statistics (Armonk, NY, USA). Data are presented as mean ± standard deviation (SD). Plasma lactate threshold values across the four-week period were analyzed using two-way repeated-measures ANOVA (within-subject factor: time; between-subject factor: group), followed by Least Significant Difference (LSD) post hoc tests for pairwise comparisons. Group-level differences in CS activity, hippocampal BDNF and GFAP levels, serum corticosterone and irisin concentrations, as well as plantaris and soleus muscle weights, were analyzed using the Kruskal–Wallis test, with Dunn’s post hoc procedure applied for pairwise contrasts following significant findings. The multivariate data structure was evaluated through principal component analysis (PCA), which was conducted using the free trial version of XLSTAT software (Addinsoft, New York, NY, USA). This analysis enabled the determination of variance distribution and separation patterns between groups based on biomarkers [33,34]. The statistical significance was set at *p* < 0.05.

## 3. Results

**Figure 2:**
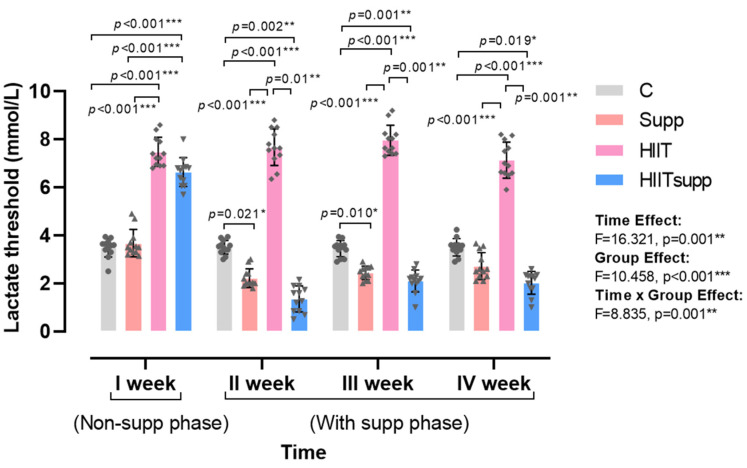
The four-week profiling of plasma lactate threshold is illustrated. Week I represented the non-supplementation phase, weeks II-IV represented the supplementation phase. Blood samples were collected 5 min post-exercise during the HIIT phase. Experimental groups are as follows: C, control group; Supp, CoQ10 supplementation group; HIIT, high-intensity interval training group; HIITsupp, high-intensity interval training combined with CoQ10 supplementation. Data are presented as mean ± SD. Statistical analysis was performed using two-way repeated-measures ANOVA (within-subject factor: time; between-subject factor: group), followed by LSD post hoc tests. Geometric symbols represent individual observations within each bar. Statistical significance is indicated by *: *p* < 0.05, **: *p* < 0.01, *** *p* < 0.001.

According to Figure 2, the main effect of time indicated a significant change in plasma lactate threshold across all groups when evaluated collectively (F = 16.321, *p* = 0.001), demonstrating that the threshold generally varied over the course of the experimental weeks. Regarding the main effect of group, a significant overall difference was observed among the groups, with the C and HIIT groups exhibiting higher lactate threshold values compared to those receiving CoQ10 supplementation (F = 10.458, *p* < 0.001). Analysis of the time × group interaction revealed that, during the non-supplementation phase, lactate threshold values were higher in the HIIT and HIITsupp groups compared with the C and Supp groups. Conversely, during the supplementation phase, the Supp and HIITsupp groups displayed lower lactate threshold values than the C and HIIT groups (Time × Group: F = 8.835, *p* = 0.001). These findings suggest that the time × group interaction was driven primarily by a pronounced reduction in lactate threshold following the supplementation phase, highlighting a statistically significant attenuating effect of CoQ10 supplementation on lactate threshold within the high-intensity interval training model, particularly in the Supp and HIITsupp groups.

**Figure 3:**
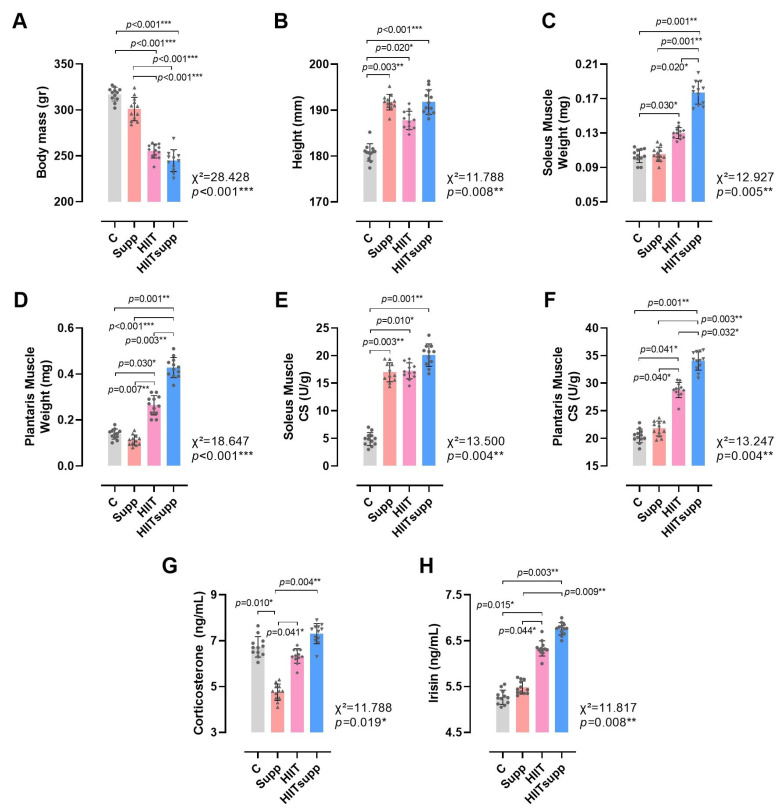
The anthropometric measurements of the experimental groups (**A**,**B**), along with the weights of the soleus muscles (**C**), the weights of the plantaris muscles (**D**), the soleus muscle CS levels (**E**), the plantaris muscle CS levels (**F**), corticosterone levels (**G**), and irisin levels (**H**) are illustrated. The experimental groups were as follows: C: control group; Supp: CoQ10 group; HIIT: high-intensity interval training; HIITsupp: high-intensity interval training combined with CoQ10 supplementation. Data are presented as mean ± SD. Statistical analysis was performed using the Kruskal–Wallis test followed by Dunn’s post hoc comparisons. Geometric symbols represent individual observations within each bar. Statistical significance is indicated by *: *p* < 0.05, **: *p* < 0.01, *** *p* < 0.001.

According to Figure 3, body mass was significantly reduced in the HIIT and HIITsupp groups compared with the C and Supp groups (Figure 3A). In contrast, body height was significantly longer in the Supp, HIIT, and HIITsupp groups compared with the C group (Figure 3B). Soleus muscle weight was higher in the HIIT group compared with the C group, with the highest value observed in the HIITsupp group; moreover, soleus muscle weight in the HIITsupp group was significantly greater than that in the C, Supp, and HIIT groups (Figure 3C). Plantaris muscle weight exhibited a similar distribution, with higher values in the HIIT group compared with the C and Supp groups, and the highest measurements again recorded in the HIITsupp group; additionally, values in the HIITsupp group were significantly greater than those in the C, Supp, and HIIT groups (Figure 3D). The CS activity in the soleus muscle was higher in the Supp, HIIT, and HIITsupp groups compared with the C group (Figure 3E), The plantaris muscle, CS activity was significantly increased in the HIIT group compared with the C and Supp groups, with the highest value observed in the HIITsupp group; furthermore, CS activity in the plantaris muscle was significantly lower in the C, Supp, and HIIT groups compared with the HIITsupp group (Figure 3F). Plasma corticosterone concentration was lower in the Supp group compared with the C, HIIT, and HIITsupp groups (Figure 3G). Plasma irisin concentration tended to be higher in the HIIT group compared with the C and Supp groups, with the highest level again observed in the HIITsupp group; additionally, plasma irisin concentration in the C and Supp groups was lower than that in the HIITsupp group (Figure 3H).

**Figure 4:**
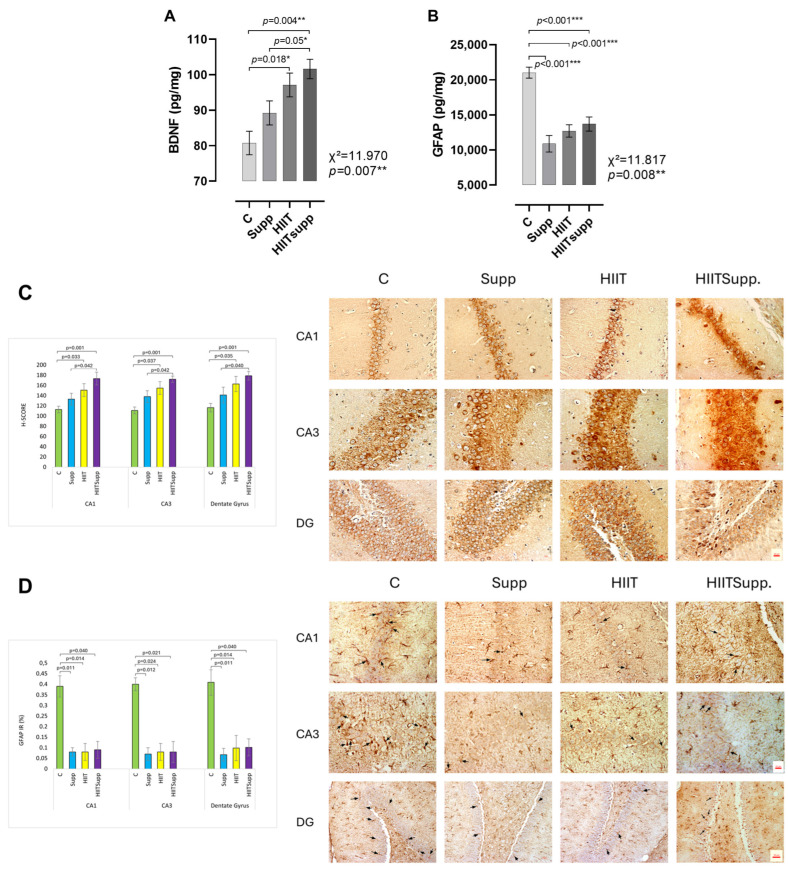
BDNF and GFAP levels in hippocampal tissue homogenates of the experimental groups, as measured by ELISA (**A**,**B**), and immunohistochemical staining with quantitative analyses of hippocampal subregions (**C**,**D**), are illustrated. BDNF immunostaining images and corresponding immunoreactivity results for the CA1, CA3, and DG subregions (**C**). Scale bar: 20 µm; magnification: 40× (for all images in (**C**)). GFAP immunostaining images of the CA1, CA3, and DG subregions. GFAP-positive astrocytes with clearly distinguishable cell bodies and processes are shown (black arrows) (**D**). Scale bars: 20 µm for CA1 and CA3 (magnification: 40×), and 50 µm for DG (magnification: 20×). The experimental groups were as follows: C: control group; Supp: CoQ10 group; HIIT: high-intensity interval training; HIITsupp: high-intensity interval training combined with CoQ10 supplementation. GFAP: glial fibrillary acidic protein; BDNF: brain-derived neurotrophic factor. Data are presented as mean ± SD. The statistical comparisons were conducted using the Kruskal–Wallis H test, followed by post-hoc Mann–Whitney U tests with Bonferroni correction. The statistical significance is indicated by *: *p* < 0.05, **: *p* < 0.01, ***: *p* < 0.001.

According to Figure 4, BDNF levels in the C group were lower than those in the HIIT group, while the HIITsupp group exhibited the highest BDNF levels among the C, Supp, and HIIT groups (Figure 4A). In contrast, GFAP levels in the C group were higher compared with the Supp, HIIT, and HIITsupp groups (Figure 4B). BDNF immunoreactivity was found to be significantly higher in both the HIIT and HIITSupp groups compared to the C group. In addition, the HIITSupp group exhibited significantly greater immunoreactivity compared to the Supp group (Figure 4C). GFAP-positive astrocytes were found to be significantly lower in the HIITSupp, HIIT, and Supp groups compared to the C group (Figure 4D).

**Figure 5:**
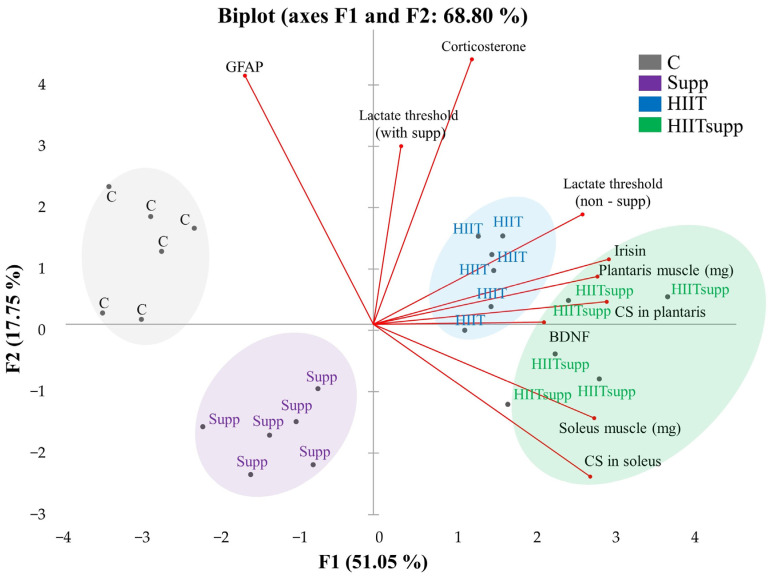
The intergroup variance distribution and separation patterns plasma and tissue samples are illustrated in the PCA biplot. The experimental groups were as follows: C: control group (black); Supp: CoQ10 group (purple); HIIT: high-intensity interval training (blue); HIITsupp: high-intensity interval training combined with CoQ10 group (green). GFAP: glial fibrillary acidic protein; BDNF: brain-derived neurotrophic factor; CS: citrate synthase levels.

According to Figure 5, Principal Component Analysis (PCA) revealed a clear variance structure that effectively distinguished the experimental groups. The first two components (F1 and F2) explained 68.8% of the total variance, while the first three components (eigenvalues ≥ 1) accounted for 83.1%. F1 (51.05%) was primarily loaded by irisin (15.6%), citrate synthase (CS) activity in the plantaris (15.3%) and soleus (13.2%) muscles, muscle weights (14.1–13.7%), and the lactate threshold (non-supplemented, 12.3%). F2 (17.75%) showed dominant loadings for corticosterone (32.7%) and GFAP (28.8%), followed by the lactate threshold (supplemented, 14.8%). Group clustering patterns in the biplot revealed distinct associations with specific biomarker vectors. The control group was aligned with GFAP and corticosterone vectors, reflecting a stress- and inflammation-dominant profile. The Supp group showed inverse loading toward these vectors, suggesting attenuation of neuroinflammatory and stress-related responses. The HIIT group was positioned near irisin, plantaris CS, and BDNF vectors, indicating strong oxidative and neurotrophic loadings under high metabolic stress. The HIITsupp group exhibited positive loadings along irisin, CS (soleus and plantaris), and BDNF vectors, while showing negative associations with GFAP and corticosterone, indicating enhanced mitochondrial and neurotrophic adaptation with reduced stress-related activity.

## 4. Discussion

Plasma lactate threshold exhibited significant effects of time, group, and their interaction across the four-week intervention. Thresholds were elevated in the HIIT and HIITsupp groups during the non-supplemented phase but declined following CoQ10 administration in both Supp and HIITsupp groups. Body mass decreased in HIIT and HIITsupp, whereas muscle mass and CS activity peaked in HIITsupp, reflecting enhanced mitochondrial adaptation. Corticosterone levels decreased in the Supp group, while irisin markedly increased in HIITsupp. PCA revealed clear separation: HIITsupp clustered with oxidative and neurotrophic markers (CS, irisin, BDNF), while the C group aligned with higher GFAP and corticosterone, indicating distinct metabolic and neural profiles. HIIT markedly reduced body mass, reflecting enhanced energy expenditure and metabolic adaptation associated with high-intensity training, whereas CoQ10 alone produced minimal change [35]. Similarly, body mass decreased in the HIIT and HIITsupp groups but remained stable in the others. Although body height increased in all groups, CoQ10 supplementation alone did not affect body weight. HIIT increases plasma lactate levels and activates mitochondrial quality control pathways [3,36]. During the non-supplemented phase, the HIIT and HIITsupp groups showed higher plasma lactate thresholds compared to the other groups. After CoQ10 supplementation, lactate thresholds decreased, with the greatest reduction observed in the HIITsupp group. PCA indicated that lactate threshold was a key parameter associated with muscle oxidative capacity.

High-intensity exercise has been demonstrated in both human and animal models to enhance mitochondrial biogenesis and oxidative capacity in skeletal muscle [37,38]. After eight weeks of intensive training, increases in CS activity in the soleus and plantaris muscles are recognized as biochemical indicators of aerobic adaptation. In the present study, CS activity in the soleus muscle was higher in the Supp, HIIT, and HIITsupp groups compared to the C group. Okamoto et al. [39] reported that high-intensity exercise increases CS activity in fast-twitch plantaris muscle, whereas moderate-intensity training primarily affects the slow-twitch soleus muscle. Similarly, Shangguan et al. [40] found that oxidative enzyme activity rises in fast-twitch fibers following high-intensity training. In this study, CS activity in the plantaris muscle was higher in the HIIT group compared to the C and Supp groups, with the highest levels observed in the HIITsupp group. PCA showed that CS activity in the plantaris and soleus muscles accounted for 15.32% and 13.21% of the variance in F1, and 10.76% in F2 for the soleus muscle. The proximity of the HIIT group to the plantaris muscle vector and the clustering of the HIITsupp group near both muscle vectors indicate a stronger mitochondrial adaptation in the HIITsupp protocol. Corticosterone, when chronically elevated, disrupts neuroendocrine balance and the stress-response system [41]. At moderate concentrations, corticosterone contributes to normal stress adaptation and maintains endocrine stability, whereas excessive levels lead to dysregulation [42]. In a post-traumatic stress disorder model, combined CoQ10 administration and treadmill exercise normalized serum corticosterone concentrations [43]. In the present study, corticosterone levels were lower in the Supp group than in the C, HIIT, and HIITsupp groups, indicating that CoQ10 modulates glucocorticoid activity [44]. Conversely, the HIIT group showed an upward trend in corticosterone, reflecting the hormonal response to high-intensity exercise. PCA identified corticosterone as the most influential variable in F2, with a loading of 32.69%.

Plasma irisin levels increased significantly in the HIIT group, with the highest response observed in the HIITsupp group. These findings indicate that high-intensity exercise effectively stimulates the release of muscle-derived myokines and that CoQ10 supplementation amplifies this effect. Puoyan Majd et al. [5] reported that eight weeks of combined HIIT and CoQ10 supplementation elevated irisin concentrations, while Amri et al. [45] found that HIIT induced a greater increase in serum irisin than endurance training. Shirvani and Arabzadeh [46] also observed that both HIIT and moderate-intensity training enhanced myokine release, with a stronger response under high-intensity conditions. In contrast, Neng Tine Kartinah et al. [47] reported no significant change in irisin levels under specific exercise protocols, suggesting that the response depends on training duration and intensity. Consistent with these findings, the HIIT and HIITsupp groups in the present study exhibited higher irisin levels and greater muscle oxidative capacity compared with the C and Supp groups, reflecting enhanced metabolic adaptation. At the mechanistic level, CoQ10 may potentiate HIIT-induced adaptations by facilitating electron transfer within the mitochondrial respiratory chain, ensuring efficient ATP production during high-intensity exercise. In addition, CoQ10 is proposed to activate Nrf2-dependent signaling pathways that complement HIIT-driven AMPK–PGC1α activation, promoting mitochondrial biogenesis and improved metabolic efficiency [3,48]

High-intensity interventions have been indicated in various human studies to significantly increase plasma BDNF levels. This increase is attributed to intermittent hypoxia during HIIT, where hypoxic responses in the motor cortex trigger BDNF expression, with similar adaptations observed in the hippocampus [49,50]. At the mechanistic level, exercise enhances neuronal activity through N-methyl-D-aspartate (NMDA) receptor activation, promoting intracellular Ca^2+^ influx and subsequent activation of the AMPK–PGC1α–FNDC5/irisin signaling cascade, a key pathway involved in BDNF synthesis and hippocampal neurogenesis [51,52,53].

BDNF concentrations were markedly higher in the Supp, HIIT, and particularly the HIITsupp groups compared with the C group, with the HIITsupp group showing the greatest increase. Similarly, BDNF immunoreactivity was elevated in both the HIIT and HIITsupp groups relative to the C group and was significantly higher in the HIITsupp group than in the Supp group. PCA indicated that BDNF contributed to 8.18% of the variance in F1 and was closely associated with muscle oxidative capacity and irisin levels, identifying the HIITsupp protocol as the most effective in promoting neurotrophic adaptations. In addition to these neurotrophic effects, exercise has been shown to modulate neuroinflammatory processes and astrocytic morphology through changes in GFAP expression [54]. Some studies report that high-intensity training increases GFAP levels [55], whereas prolonged and balanced regimens suppress glial activation and exert neuroprotective effects [56,57]. These regulatory effects are primarily mediated through the suppression of NF-κB–driven pro-inflammatory signaling and the activation of Nrf2-dependent antioxidant pathways, which collectively reduce astrocytic GFAP expression and maintain redox homeostasis [58,59]. Notably, Pin-Barre and Constans [60] demonstrated that HIIT reduced GFAP-positive cell counts in a middle cerebral artery occlusion model, thereby limiting inflammatory responses. In the present study, GFAP levels were highest in the control group and lower in the Supp, HIIT, and HIITsupp groups. PCA identified GFAP and corticosterone as dominant loadings defining the control group, whereas the HIITsupp group clustered with BDNF, muscle CS activity, and irisin, reflecting coordinated neurotrophic and anti-inflammatory adaptations. CoQ10-supported HIIT appears to enhance these effects through complementary mechanisms, promoting BDNF-related neuroplasticity via the AMPK–PGC1α–FNDC5/irisin axis while suppressing GFAP-mediated astrocytic activation through Nrf2-dependent redox regulation [15]. The concurrent modulation of BDNF and GFAP in the HIITsupp group underscores the integration of metabolic and neural adaptive pathways that may strengthen resilience under physiological or stress-related conditions. If validated in humans, such mechanisms could inform strategies to preserve performance capacity and mitigate functional decline associated with aging or neurodegenerative disorders [5,61].

### Strengths and Limitations

This study used a randomized, repeated-measures, post-test-controlled design to ensure methodological rigor. The standardized HIIT protocol and CoQ10 supplementation enabled assessment of their independent and combined effects on skeletal muscle and hippocampal markers. Multimodal analyses, including biochemical assays, immunohistochemistry, and PCA, provided an integrated evaluation of metabolic and neuroendocrine responses. However, the use of only young male rats limits generalizability. The fixed dose and short duration may not capture long-term or dose–response effects, and the lack of cognitive testing restricts functional interpretation. Future studies with extended protocols, variable dosing, and inclusion of both sexes are recommended to enhance translational relevance.

## 5. Conclusions

This study demonstrated that the combined HIIT and CoQ10 protocol induced stronger physiological and neurobiological adaptations than either intervention alone. HITT with CoQ10 supplementation modulated lactate threshold dynamics, enhanced muscle oxidative capacity and mitochondrial function, and reduced corticosterone and GFAP levels, indicating lower stress and neuroinflammatory responses. The HIITsupp group also demonstrated the highest hippocampal BDNF concentrations and immunoreactivity. PCA results supported these findings, revealing coordinated changes across metabolic, hormonal, and neurotrophic markers in the HIITsupp group.

## Data Availability

The original contributions presented in this study are included in the article. Further inquiries can be directed to the corresponding author.

## Abbreviations

The following abbreviations are used in this manuscript:

AMPK	AMP-activated protein kinase
BDNF	Brain-Derived Neurotrophic Factor
CS	Citrate Synthase
CoQ10	Coenzyme Q10
GPR81	G-protein-coupled receptor 81
GFAP	Glial Fibrillary Acidic Protein
MICT	Moderate Intensity Continuous Training
MCTs	Monocarboxylate Transporters
NMDA	N-methyl-D-aspartate receptor activation
Nrf2	Nuclear factor erythroid 2-related factor-2
NQO1	NAD(P)H: quinone oxidoreductase-1
NQO1	Peroxisome proliferator-activated receptor gamma coactivator 1-alpha
ROS	Reactive Oxygen Species
TCA	Tricarboxylic Acid
HO-1	Heme oxygenase-1
HICT	High-Intensity Continuous Training
HIIT	High-Intensity Interval Training
PCA	Principal Component Analysis
